# miR-374a Regulates Inflammatory Response in Diabetic Nephropathy by Targeting MCP-1 Expression

**DOI:** 10.3389/fphar.2018.00900

**Published:** 2018-08-10

**Authors:** Zijun Yang, Zuishuang Guo, Ji Dong, Shifeng Sheng, Yulin Wang, Lu Yu, Hongru Wang, Lin Tang

**Affiliations:** ^1^Department of Nephropathy, The First Affiliated Hospital of Zhengzhou University, Zhengzhou, China; ^2^Henan Medical College, Zhengzhou, China

**Keywords:** miR-374a, diabetic nephropathy, MCP-1, inflammatory response, high glucose

## Abstract

The microRNA (mir)-374a has been implicated in several types of human cancer; however, its role in diabetic nephropathy (DN) remains unclear. Monocyte chemoattractant protein (MCP)-1 is a chemokine that recruits macrophages to inflammatory sites and is important for the development and progression of DN. However, the relationship between miR-374a and MCP-1 in DN is unknown. We addressed this in the present study by examining the expression of these factors in kidney tissue samples from DN patients and through loss- and gain-of-function experiments using HK2 human renal tubular epithelial cells. We found that miR-374a was downregulated whereas MCP-1 was upregulated in DN tissue. A bioinformatics analysis revealed that MCP-1 is a putative target of miR-374a. To confirm this relationship, HK2 cells treated with normal glucose (5.6 mmol/l D-glucose), high glucose (HG) (30 mmol/l D-glucose), or high osmotic pressure solution (5.6 mmol/l D-glucose + 24.4 mmol/l D-mannitol) were transfected with miR-374a mimic or inhibitor. miR-374a mimic reduced MCP-1 mRNA expression and migration of co-cultured U937 cells, whereas miR-374a inhibition had the opposite effects. Additionally, interleukin-6 and -18 and tumor necrosis factor-α levels were downregulated by transfection of miR-374a mimic. On the other hand, MCP-1 overexpression reversed the inhibitory effects of miR-374a in HK2 cells. Thus, miR-374a suppresses the inflammatory response in DN through negative regulation of MCP-1 expression. These findings suggest that therapeutic strategies that target the miR-374a/MCP-1 axis can be an effective treatment for DN.

## Introduction

Diabetic nephropathy (DN) is one of the major microvascular complications associated with diabetic patients. Approximately 15–25% of type 1 diabetes and 30–40% of type 2 diabetes develop DN, which is the main cause of end-stage renal disease (ESRD) (Schernthaner and Schernthaner, 2013). The United States Renal Data System reported that type 2 DN accounts for 35–50% of ESRD cases (Collins et al., 2014). There are currently no treatments for DN other than symptomatic relief such as blood pressure and glucose control, administration of renin–angiotensin–aldosterone inhibitors, and dialysis and kidney transplantation. None of these treatments has reduced the high morbidity and mortality rates associated with DN, resulting in a significant health care burden for society (Lopez-Vargas et al., 2016). Patients undergoing dialysis have a mortality rate of 20% per year, and transplantation is restricted by the lack of renal allografts (DeFronzo et al., 2017). Clarifying the mechanism underlying DN and developing novel and effective therapeutic strategies is critical for improving disease outcome.

Recent studies have demonstrated an important role for inflammation in the development and progression of DN (Feng et al., 2015; Sancar-Bas et al., 2015; Zheng and Zheng, 2016). Glomerular sclerosis and interstitial fibrosis are the major pathological changes associated with DN, and renal biopsies suggest that macrophage infiltration and elevated levels of inflammatory cytokines are closely associated with kidney fibrosis. Inflammatory factors can activate myofibroblasts at injury sites in the kidney while inducing the differentiation of mesangial cells, glomeruli, and renal tubular epithelial cells into fibroblasts, resulting in enhanced extracellular matrix (ECM) production and deposition, which in turn promote tubulointerstitial fibrosis (Kanasaki et al., 2013; Maeshima et al., 2014).

Elevated levels of monocyte chemoattractant protein (MCP)-1 in type 1 and 2 DN patients have been linked to DN development (Banba et al., 2000) through recruitment of macrophages and monocytes to inflammatory sites and upregulation of cytokines such as interleukin (IL)-1, -6, -8, and tumor necrosis factor (TNF)-α. MCP-1 expression is induced by hyperglycemia, lipid metabolism, advanced glycation end products, overstimulation of the renin-angiotensin system, oxidative stress, and nuclear factor (NF)-κB signaling (Wei et al., 2015) in DN patients.

MicroRNAs (miRNAs) are endogenous non-coding RNAs 20–25 nucleotides in length that negatively regulate gene expression in animals and plants by targeting the 3′ untranslated region (3′-UTR) of target mRNA (Cai et al., 2013). Aberrant miRNA expression has been reported in several kidney conditions including DN, polycystic kidney disease, renal fibrosis, drug-induced kidney injury, and kidney transplantation (Cardenas-Gonzalez et al., 2017; Celen et al., 2017; Cho et al., 2017; Huttenhofer and Mayer, 2017; Wonnacott et al., 2017; Yheskel and Patel, 2017; Zununi et al., 2017). miRNAs participate in positive or negative feedback loops by targeting NF-κB, IκB, inhibitor of IκB kinase (IKK) (Ma et al., 2011). Members of the miR-184, -29, and -200 families as well as miR-192 and miR-21 have also been implicated in fibrotic processes in DN (Kato and Natarajan, 2014, 2015; McClelland et al., 2014; Rudnicki et al., 2015; Zanchi et al., 2017).

miR-374a has been reported to be involved in many types of cancer. For example, miR-374a suppresses lung adenocarcinoma cell proliferation and invasion by targeting transforming growth factor (TGF)-α gene expression (Wu et al., 2016), whereas in gastric cancer, miR-374a level is increased and targets SRC kinase signaling inhibitor 1 to promote cell proliferation, migration, and invasion (Xu et al., 2015). miR-374a also negatively regulates WNT5A, Wnt inhibitory factor 1, and phosphatase and tensin homolog to promote breast cancer epithelial-to-mesenchymal transition and metastasis *in vitro* and *in vivo* (Cai et al., 2013). However, the molecular mechanism and function of miR-374a in DN is not known.

We addressed this in the present study by examining the expression of miR-374a and MCP-1 in kidney tissue samples from DN patients and performing loss- and gain-of-function experiments using HK2 human renal tubular epithelial cells. We found that miR-374a is downregulated in DN tissues and HK2 cells treated with high glucose (HG). We also confirmed that miR-374a suppresses the production of cytokines including IL-6 and -18, TNF-α, and MCP-1. These results indicate that miR-374a inhibits the inflammatory response via modulation of MCP-1 during DN progression.

## Materials and Methods

### Clinical Samples and Immunohistochemistry

Human DN (*n* = 10) and adjacent non-cancerous (*n* = 5) tissue samples (3–5 cm from the tumor edge) were obtained from patients without diabetes mellitus or any other type of kidney disease who underwent surgical resection for kidney tumors at the First Affiliated Hospital of Zhengzhou University. The clinical characteristics of DN patients are shown in **Supplementary Table 1**. This study was approved by the Ethics Committee of the First Affiliated Hospital of Zhengzhou University. Kidney tissue sections approximately 4 μm thick and embedded in paraffin were labeled with an antibody against MCP-1 using a commercial kit (Abcam, Cambridge, MA, United States). Brown positive staining in DN (*n* = 10) and adjacent non-cancerous tissue (*n* = 5) was semi-quantitatively scored based on density and area by an independent investigator in a blinded fashion.

### Hematoxylin and Eosin (HE) Staining

Kidney tissue samples were immersed in 4% paraformaldehyde for 4 h and then transferred to 70% ethanol. Individual lobes of renal biopsy specimens were placed in processing cassettes, dehydrated through a graded series of alcohol, and embedded in paraffin. The renal tissue blocks were cut into sections 4 μm thick that were deparaffinized in xylene, rehydrated with decreasing concentrations of ethanol, washed in phosphate-buffered saline, and stained with HE. The sections were then dehydrated in increasing concentrations of ethanol and xylene and mounted for microscopic observation.

### Plasmid Construction and Luciferase Reporter Assay

The 3′-UTR sequence of MCP-1 was predicted to interact with miR-374a by bioinformatics analysis with TargetScan, Microrna, and PicTar programs. Mutant (MT) and wild-type (WT) MCP-1 3′-UTR sequences were synthesized and inserted into the pmirGLO vector. pmirGLO-WT-MCP-1-3′-UTR or pmirGLO-MT-MCP-1-3′-UTR constructs were co-transfected into 293T cells with miR-374a mimic or a scrambled sequence (negative control). After 48 h, firefly and Renilla luciferase activities were measured using the Dual Luciferase Reporter Assay kit (Promega, Beijing, China) according to the manufacturer’s instructions. The assay was performed in triplicate.

### Cell Culture and Treatment

HK2 or U937cells (Chinese Academy of Sciences Shanghai Cell Bank, Shanghai, China) were cultured in Dulbecco’s modified Eagle’s medium (DMEM) supplemented with 5.6 mmol/l glucose, 10% fetal bovine serum, 100 U/ml penicillin, and 100 μg/ml streptomycin in a 5% CO_2_ incubator at 37°C. The cells were trypsinized and seeded in 6-well culture plates at a density of 1 × 10^6^/ml and grown to over 80% confluence; cultures were synchronized with serum-free medium for 12 h and used for experiments when they reached 70–80% confluence. HK2 cells were treated with normal glucose (NG; 5.6 mmol/l D-glucose), HG (30 mmol/l D-glucose), or high osmotic pressure solution (HO; 5.6 mmol/l D-glucose + 24.4 mmol/l D-mannitol).

### Quantitative Real-Time (qRT)-PCR

Total RNA was extracted and reverse transcribed into cDNA that was used as a template for qRT-PCR on a DNA Engine Opticon system (Fuzhong Bio-Company, Shanghai, China) in 96-well plates under the following reaction conditions: 95°C for 10 min, and 40 cycles at 95°C for 15 s and 60°C for 1 min. Glyceraldehyde 3-phosphate dehydrogenase (GAPDH) was used as an internal control to determine the relative expression levels of target genes; U6 small nuclear RNA served as an endogenous control for miR-374a. The 2^-ΔΔCt^ method was used to normalize and calculate fold changes in gene expression.

### Western Blot Analysis

Cells were harvested and lysed in radioimmunoprecipitation assay buffer containing protease and phosphatase inhibitors and centrifuged at 12,000 rpm for 20 min at 4°C. Protein concentration was measured with the bicinchoninic acid assay (BCA). Briefly, 20 μl of protein sample were added to 200 μl BCA regent, and absorbance at 590 nm was measured on a microplate reader. A standard curve was generated from the measured values and used to determine protein content. Protein samples in loading buffer were boiled at 100°C for 5 min and resolved by 12% sodium dodecyl sulfate polyacrylamide gel electrophoresis, and electrotransferred to a polyvinylidene difluoride membrane that was blocked with 5% skimmed milk and probed with a primary antibody against MCP-1 (Abcam; 1:500) for 12 h at 4°C. After washing, the membrane was incubated with an appropriate secondary antibody at room temperature for 2 h; 3′-diaminobenzidine was used for visualization. The ChemiDoc MP Imaging System (Bio-Rad, Shanghai, China) was used for densitometric analysis. GAPDH (Santa Cruz Biotechnology, Santa Cruz, CA, United States; 1:1000) was used as the loading control.

### Enzyme-Linked Immunosorbent Assay (ELISA)

After establishing the co-culture system, cells were cultured under the various treatment conditions. IL-6 and -18 and TNF-α levels in the culture supernatant were measured by ELISA using the appropriate ELISA kit (R&D Systems, Minneapolis, MN, United States) according to the manufacturer’s instructions.

### Transwell Migration Assay

Corning 24-well Transwell co-culture plates (pore size: 5 μm) were used to evaluate cell migration. The co-culture system was divided into two compartments separated by a microporous membrane that permitted diffusion of soluble molecules and chemotactic agents and the interaction of cells in the different layers. Before the experiment, HK2 cells were inoculated in the lower compartment at a density of 1 × 10^5^ cells/ml, while 600 μl culture medium were added to the lower chamber. Cells were synchronized with serum-free DMEM/F12 for 12 h before the experiment. U937 cells were inoculated into the upper chamber at a density of 5 × 10^4^ cells/ml; 200 μl of culture medium were added to the cells, which were synchronized 12 h before the experiment. The cells remaining on the upper surface of the membrane were removed with a cotton swab, and those that had migrated through the 5-μm-diameter pores and had adhered to the lower surface of the membrane were fixed with 4% paraformaldehyde, stained with crystal violet, and photographed under a light microscope.

### Cell Transfection

HK2 cells were seeded in 6-well plates. When they reached 50–80% confluence, they were transfected with miR-374a mimic or inhibitor (GenePharma, Shanghai, China) using Lipofectamine 2000 (Invitrogen, Carlsbad, CA, United States) according to the manufacturer’s instructions. After 6 h, the cells were treated with NG, HG, or HO. Protein and RNA were extracted for analyses.

### Overexpression of MCP-1

The pcDNA3.1-MCP-1 vector lacking the 3′-UTR was constructed and transfected into HK2 cells using Lipofectamine 2000.

### Statistical Analysis

Data are expressed as mean ± SD. Means of multiple groups were compared by one-way analysis of variance. The statistical significance of differences between two groups was evaluated with the least significant difference test. *P* < 0.05 was considered statistically significant. The relationship between two variables was evaluated by Spearman’s correlation analysis. Statistical analyses were performed using SPSS v.18.0 software (SPSS Inc., Chicago, IL, United States).

## Results

### HG Induces MCP-1 but Suppresses miR-374a Expression

**FIGURE 1:**
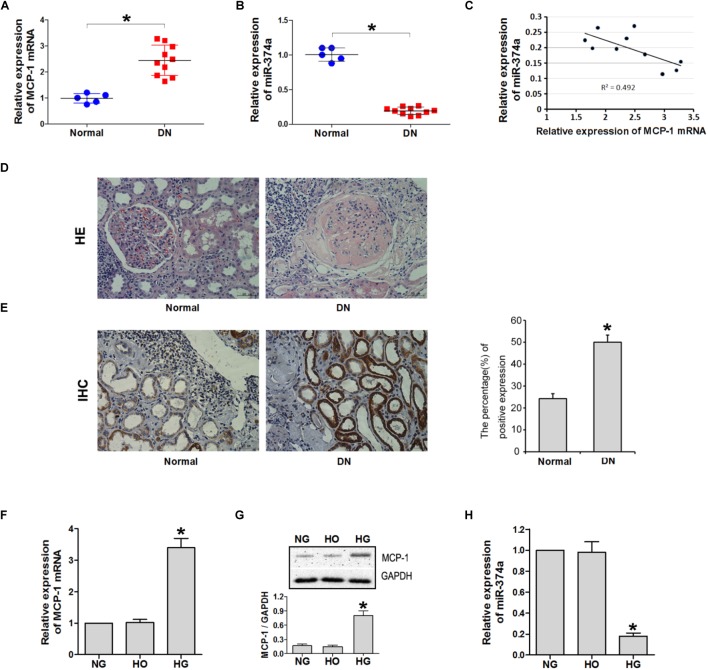
MCP-1 and miR-374a expression in DN tissues. qRT-PCR analysis of MCP-1 mRNA **(A)** and miR-374a **(B)** levels in DN tissues. **(C)** Spearman’s correlation analysis demonstrating an inverse correlation between miR-374a and MCP-1 mRNA levels. ^∗^*P* < 0.05. **(D)** Histological analysis of normal (*n* = 5) and DN (*n* = 10) tissues, as determined by HE staining. **(E)** Upregulation of MCP-1 in DN tissue, as determined by immunohistochemistry. HG induced MCP-1 but suppressed miR-374a in HK2 cells. **(F)** qRT-PCR and **(G)** western blot analysis of MCP-1 expression in HK2 cells treated with NG, HG, or HO for 24 h. ^∗^*P* < 0.05 vs. NG or HO group (*n* = 3). **(H)** miR-374a expression in each group as determined by qRT-PCR. ^∗^*P* < 0.05 vs. NG group (*n* = 3).

MCP-1 mRNA and miR-374a levels were detected in DN (*n* = 10) and adjacent non-cancerous (*n* = 5) tissues by qRT-PCR. MCP-1 was upregulated whereas miR-374a was downregulated in DN relative to control tissue (**Figures 1A,B**); there was a negative linear correlation between their expression levels (**Figure 1C**). A histological analysis revealed that DN tissue had abnormal architecture, as evidenced by glomerular swelling and vacuolization in the endothelial lining (**Figure 1D**). An immunohistochemical analysis confirmed upregulation of MCP-1 in DN tissue (**Figure 1E**). MCP-1 and miR-374a levels in the supernatant of HK2 cell cultures incubated with NG, HG, and HO were evaluated by qRT-PCR and western blotting. After 24 h of HG stimulation, MCP-1 mRNA and protein levels were almost 3-fold higher than in the NG or HO group (**Figures 1F,G**). In contrast, miR-374a was downregulated approximately 5 fold in the HG as compared to the NG or HO group after 24 h (**Figure 1H**). These results suggest a regulatory relationship between MCP-1 and miR-374a in DN.

### MCP-1 Is a Direct Target of miR-374a

**FIGURE 2:**
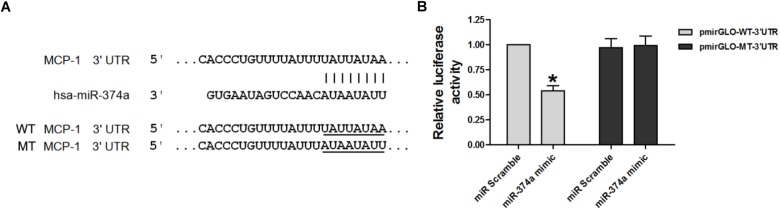
miR-374a negatively regulates MCP-1. **(A)** WT and MT complementary sequences of MCP-1 3′-UTR are shown with the miR-374a sequence. **(B)** Luciferase reporter assay showing the inhibitory effect of miR-374a on MCP-1 3′-UTR luciferase activity in 293T cells transfected with miR-374a mimic. ^∗^*P* < 0.05.

Given the negative correlation between the expression levels of miR-374a and MCP-1, we investigated whether MCP-1 is a target of miR-374a regulation. A bioinformatics analysis with TargetScan, Microrna, and PicTar programs identified a short complementary sequence shared by miR-374a and MCP-1 (**Figure 2A**). We carried out a luciferase reporter assay to determine whether miR-374a regulates MCP-1 expression by transfecting WT-MCP-1-3′UTR and MT-MCP-1-3′UTR plasmid constructs into 293T cells along with a miR-374a mimic or scrambled control sequence. The results showed that miR-374a mimic suppressed the transcriptional activity of the luciferase-WT-MCP-1-3′-UTR reporter by approximately 50% relative to the control group. However, the activity of MT-MCP-1-3′UTR harboring a mutated miR-374a binding site was not suppressed by miR-374a mimic (**Figure 2B**). These results indicate that miR-374a inhibits MCP-1 expression through a direct regulatory mechanism.

### HG Induces U937 Cell Migration and Expression of Inflammatory Cytokines via miR-374a

**FIGURE 3:**
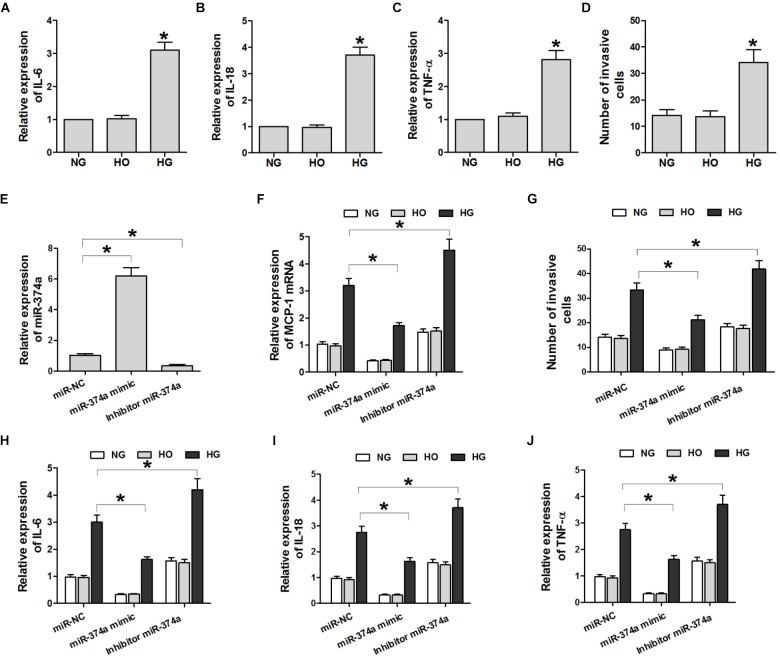
HG induces U937 cell migration and inflammatory cytokine expression through miR-374a. **(A–C)** ELISA analysis of IL-6 and -18 and TNF-α in the culture supernatant. ^∗^*P* < 0.05 vs. NG group (*n* = 3). **(D)** Quantitative analysis of U937 cell migration in different groups. ^∗^*P* < 0.05 vs. NG or HO group (*n* = 3). **(E,F)** qRT-PCR analysis of miR-374a and MCP-1 mRNA. ^∗^*P* < 0.05 vs. negative control (miR-NC) group (*n* = 3). **(G)** Quantification of U937 cells with the transwell migration assay. ^∗^*P* < 0.05 vs. miR-NC group (*n* = 3). **(H–J)** After the transwell migration assay, IL-6 and -18 and TNF-α levels in the culture supernatant of HK2 cells transfected with a miR-374a mimic or inhibitor were monitored. ^∗^*P* < 0.05 vs. miR-NC group (*n* = 3).

To determine whether miR-374a suppresses the inflammatory response in DN by targeting MCP-1 expression, we carried out a transwell migration assay with U937 and HK2 cells seeded in the top and bottom chambers, respectively. The cells were treated with NG, HG, or HO and IL-6 and -18 and TNF-α levels in the supernatant were determined by ELISA and the number of U937 cells in the supernatant was counted. IL-6 and -18 and TNF-α levels were higher in the HG group than in the NG and HO groups (**Figures 3A–C**). In addition, HK2 cells treated with HG induced the migration of U937 cells as compared to the NG and HO groups (**Figure 3D**). Thus, HG induces U937 cell migration and promotes inflammatory cytokine expression.

To investigate the effect of miR-374a on the production of inflammatory cytokines in U937 cells, miR-374a mimic or inhibitor was overexpressed in HK2 cells. The inhibitor significantly reduced miR-374a expression (**Figure 3E**). Compared to the negative control group, miR-374a mimic suppressed the HG-induced upregulation of MCP-1 transcript and IL-6 and -18 and TNF-α proteins, whereas miR-374a inhibitor had the opposite effect (**Figures 3F,H–J**). Similarly, miR-374a mimic suppressed HG-induced migration of U937 cells, which was reversed by miR-374a inhibitor (**Figures 3F,G**). These data suggest that increased miR-374a expression suppresses the inflammatory response in DN.

### MCP-1 Overexpression Antagonizes the Effect of miR-374a Mimic

**FIGURE 4:**
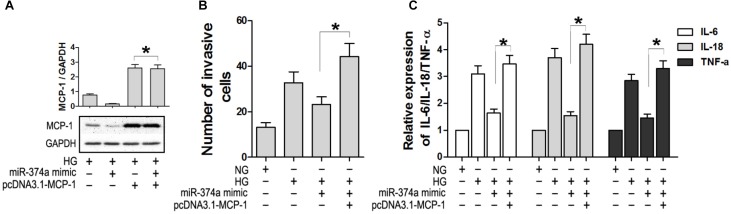
Overexpression of MCP-1 antagonizes the effect of miR-374a mimic. **(A)** MCP-1 protein level in HK2 cells after co-transfection of pcDNA3.1-MCP-1, as determined by western blotting. ^∗^*P* < 0.05. **(B)** Numbers of migrating U937 cells in the co-culture system with or without MCP-1 overexpression under HG conditions. **(C)** Detection of IL-6 and -18 and TNF-α by ELISA in the co-culture system with or without MCP-1 overexpression. ^∗^*P* < 0.05.

To confirm the regulatory relationship between miR-374a and MCP-1, we overexpressed MCP-1 lacking the 3′-UTR in HK2 cells transfected with miR-374a mimic (**Figure 4A**). MCP-1 overexpression was not suppressed by miR-374a mimic (**Figure 4A**). Furthermore, the migration of U937 cells was not blocked under HG conditions upon co-transfection of pcDNA3.1-MCP-1 and miR-374a mimic (**Figure 4B**), which also did not affect the production of IL-6 and-18 and TNF-α in the HK2 and U937 cell co-culture system with HG (**Figure 4C**). These results confirm that miR-374a negatively regulates MCP-1 expression through binding to the 3′-UTR region.

## Discussion

In this study, we demonstrated that miR-374a expression was lower in DN as compared to normal tissues and is negatively correlated with that of MCP-1. Overexpression of miR-374a suppressed MCP-1 expression; IL-6 and -18 and TNF-α production; and cell invasion, whereas miR-374a inhibition had the opposite effects. The results of the luciferase reporter assay showed that miR-374a directly targets the 3′-UTR of MCP-1 transcript. On the other hand, MCP-1 overexpression antagonized the inhibitory effect of miR-374a mimic on the inflammatory response, suggesting that MCP-1 is a downstream target of miR-374a during DN. This is the first report describing miR-374a function and its relationship to MCP-1 in DN.

Previous studies have reported that miRNAs regulate gene expression during DN progression (Long et al., 2010; Wang et al., 2010) by targeting signaling pathways related to mechanical stress, oxidative stress, generation of advanced glycation end products and their receptors, renin–angiotensin–aldosterone system (RAAS) activation, and autophagy (Wang et al., 2008; Macconi et al., 2012; Fiorentino et al., 2013; Li et al., 2013; Tekirdag et al., 2013). For example, miR-184 was shown to promote tubulointerstitial fibrosis as a downstream effector of albuminuria through lipid phosphate phosphatase 3 (Zanchi et al., 2017). Hyperglycemia influenced pathogenic processes during DN through an miR-23b/GTPase activating protein (SH3 domain)-binding protein 2 feedback circuit involving p38 mitogen-activated protein kinase (MAPK) and p53 (Zhao et al., 2016). Proteinuria, genetics, ethnicity, hypoxia, ischemia, and inflammation have been proposed as factors contributing to DN (Fernandez et al., 2012). miRNAs also regulate inflammation by targeting TGF-β-activated kinase 1/MAPK kinase kinase 7-binding protein 2/3, and inhibitor of NF-κB kinase subunit-α (Zhu et al., 2012), while miR-155 and -146 were shown to regulate inflammatory responses through NF-κB signaling (Mann et al., 2017). Our results also provide evidence for the involvement of miR-374a in the inflammatory response, although the detailed mechanism remains to be elucidated.

MCP-1 is a ligand of CC chemokine receptor 2 that acts as a chemotactic factor for monocytes/macrophages and activated T cells (Boels et al., 2017). MCP-l levels in peripheral blood of diabetes mellitus patients were positively correlated with urinary albumin excretion rate, and MCP-l mRNA and protein levels are higher in DN than in normal renal tissue (Giunti et al., 2010). There are several mechanisms by which MCP-1 may contribute to DN. Firstly, direct stimulation by HG could lead to upregulation of MCP-1. Secondly, the general existence of blood lipid metabolism disorder in DN and high levels of low-density lipoprotein and its metabolite could induce MCP-1 production by mesangial cells (Rutledge et al., 2010). Thirdly, endothelial and mesangial cells may produce MCP-1 in response to IL-l, TNF-α, and platelet-derived growth factor stimulation (Fufaa et al., 2015). Fourthly, activated RAAS could regulate MCP-1 by increasing macrophage infiltration and ECM accumulation through NF-κB signaling (Yang et al., 2016).

In this study, we confirmed the regulation of MCP-1 by miR-374a in HK2 cells, which suggests an important role for this signaling pathway in the development and progression of DN. In cells expressing miR-146a and -146b, IκB phosphorylation on serine 32–which is essential for its degradation—was reduced to 40 or 20% of control levels in an experiment that directly demonstrated the negative regulation of NF-κB by miR-146 (Ma et al., 2011). Advanced oxidation protein product-induced MCP-1 expression has been linked to the IKK/NF-κB signaling pathway (Zhao et al., 2015). Based on these findings, we propose that miR-374a interacts with MCP-1 via the NF-κB signaling pathway in DN.

## Conclusion

Our results indicate that MCP-1 is a direct downstream target of miR-374a in DN. Thus, therapeutic strategies targeting this axis may be effective for the treatment of DN.

## Ethics Statement

This study was carried out in accordance with the recommendations of the Institutional Review Board and Ethics Committee of the First Affiliated Hospital of Zhengzhou University, with written, informed consent from all subjects in accordance with the Declaration of Helsinki. The protocol was approved by the Institutional Review Board and Ethics Committee of the First Affiliated Hospital of Zhengzhou University.

## Author Contributions

LT conceived and designed the study. SS, YW, LY, and HW performed the experiments. JD analyzed the data. ZY and ZG wrote the paper and contributed equally to this work. LT and JD reviewed and edited the manuscript. All authors read and approved the final manuscript.

## Conflict of Interest Statement

The authors declare that the research was conducted in the absence of any commercial or financial relationships that could be construed as a potential conflict of interest.
