# Supplementary figures and images for: miR-374a Regulates Inflammatory Response in Diabetic Nephropathy by Targeting MCP-1 Expression
Source: Front Pharmacol. 2018 Aug 10;9:900. doi: 10.3389/fphar.2018.00900 (PMC6095963; doi:10.3389/fphar.2018.00900)

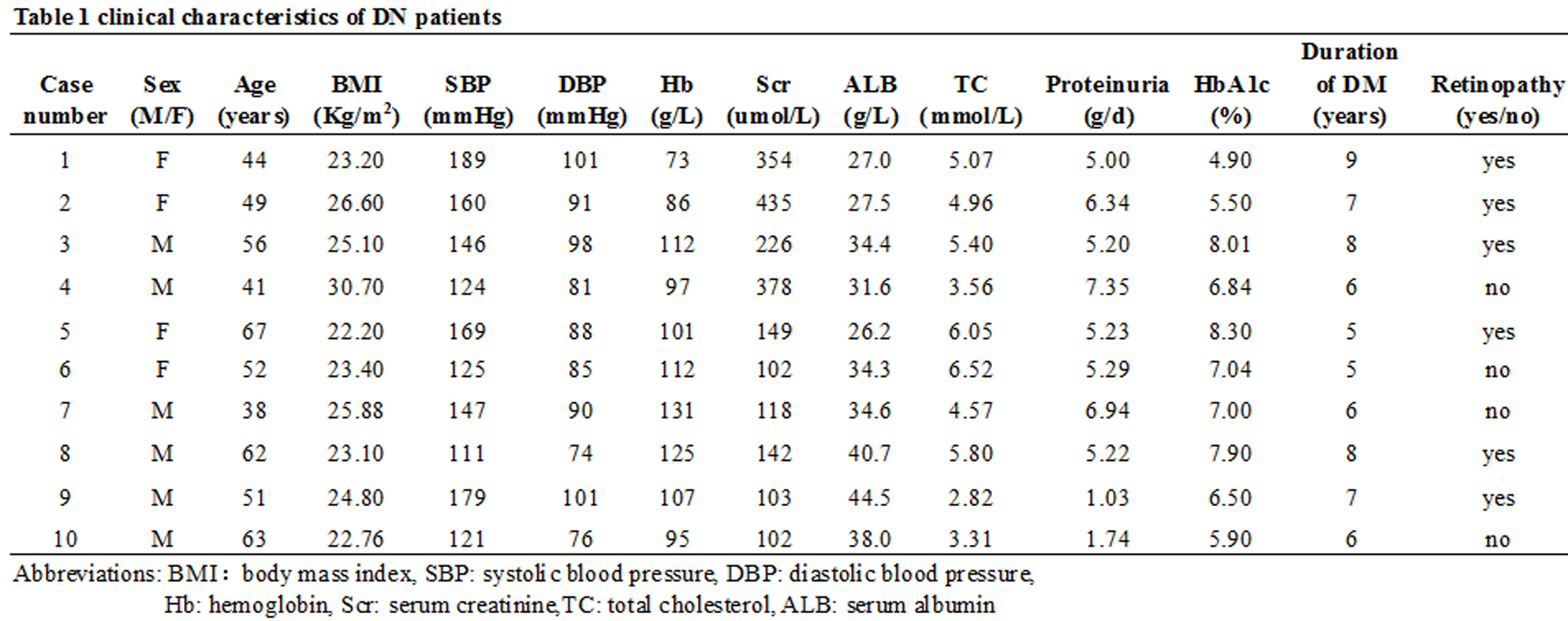

Supplement: Supplementary file 1 [file Image_1.JPEG]
